# Validation of the geometric equivalent field concept in total scatter factor calculations, for half‐, quarter‐ and off‐isocenter asymmetric square fields

**DOI:** 10.1002/acm2.14103

**Published:** 2023-11-13

**Authors:** Mohammad Samir Hmodi, Majeda Nahili, Ioannis A. Tsalafoutas, Bassam Saad, Ali Hasan, Ousamah Anjak, Karlos Shamout

**Affiliations:** ^1^ Department of Physics Faculty of Sciences Damascus University Damascus Syria; ^2^ Department of Oncology Tishreen University Hospital Lattakia Syria; ^3^ Medical Physics Section OHS Department Hamad Medical Corporation Doha Qatar; ^4^ Cancer Research Center Tishreen University Hospital Lattakia Syria; ^5^ Department of Protection and Safety Atomic Energy Commission of Syria Damascus Syria

**Keywords:** asymmetric square fields, equivalent fields, monitor unit verification, off‐axis ratio, total scatter factor

## Abstract

**Objective:**

Monitor unit (MU) verification for any symmetric or asymmetric field is performed using a total scatter factor (S_cp_), that is calculated based on the geometric equivalent square field (GESF) concept. In this study, we measured the S_cp_ of various asymmetric square fields (ASF_s_) and their respective GESFs.

**Methods:**

Square half‐fields (SHF_s_), square quarter‐fields (SQF_s_) and square off‐isocenter fields (SOF_s_), with sizes ranging from 3×3 cm^2^ to 20×20 cm^2^ were created, by varying the collimator jaws of two Varian iX Linacs (6/18 and 6/23 MV). A semi‐flex ion chamber was used to measure S_cp_ at a depth of 10 cm within a water phantom, at the effective field center (EFC) of all ASF_s_, and at the isocenter (IC) of their respective GESFs. The later S_cp_ values were corrected by the off‐axis ratio [OAR(r)] of the 40×40 cm^2^ field size, where *r* is the distance between EFC and IC.

**Results:**

The results show that the S_cp_ (EFC) is independent of the type of the ASF (SHF, SQF, or SOF) and no significant difference exists between the 18 and 23 MV beams. Compared with the S_cp_ (IC), the S_cp_ (EFC) increased with increasing *r*, by up to 2% and 4% for 18/23 and 6 MV, respectively.

**Conclusions:**

The GESF concept provides acceptable accuracy (< 2%) for the calculation of S_cp_ of the ASFs used in most clinical situations (except from SOF with EFC at large *r*), and thus can be used in MU verification calculations.

## 1 INTRODUCTION

The total scatter factor (S_cp_), also referred to as output factor in water, has been always an important parameter for the calculation of monitor units (MUs) in radiotherapy, as a function of the treatment field (TF) size. Two different approaches have been used to calculate the S_cp_. In the first approach, which is also applicable in irregular TFs, S_cp_ is derived by the product of two factors, S_c_ and S_p_.^1^, ^2^ S_c_ is the head or collimator scatter factor, which is related to the collimator opening dimensions. S_c_ accounts for the increase in output observed with increased field size, attributed to the increased amount of radiation scattered by the collimator jaws that reaches the phantom (or the patient). S_p_ is the phantom scatter factor, which is related to the amount of scatter produced within the irradiated volume of the phantom/patient. For the S_p_ calculations it is taken into account that the presence of blocks or a multi‐leaf collimator (MLC), modify the dimensions of the TF and therefore the amount of scattered radiation produced within the phantom/patient. In the second approach, the S_cp_ is taken directly from tables, for different field sizes (collimator dimensions) and beam energies,^3^ and incorporates both the head and phantom scatter. However, this approach is applicable in manual MU calculations for regular treatment fields only (when no blocks are used) or for a quick cross‐check of the MU calculated by a treatment planning system (TPS).

**FIGURE 1:**
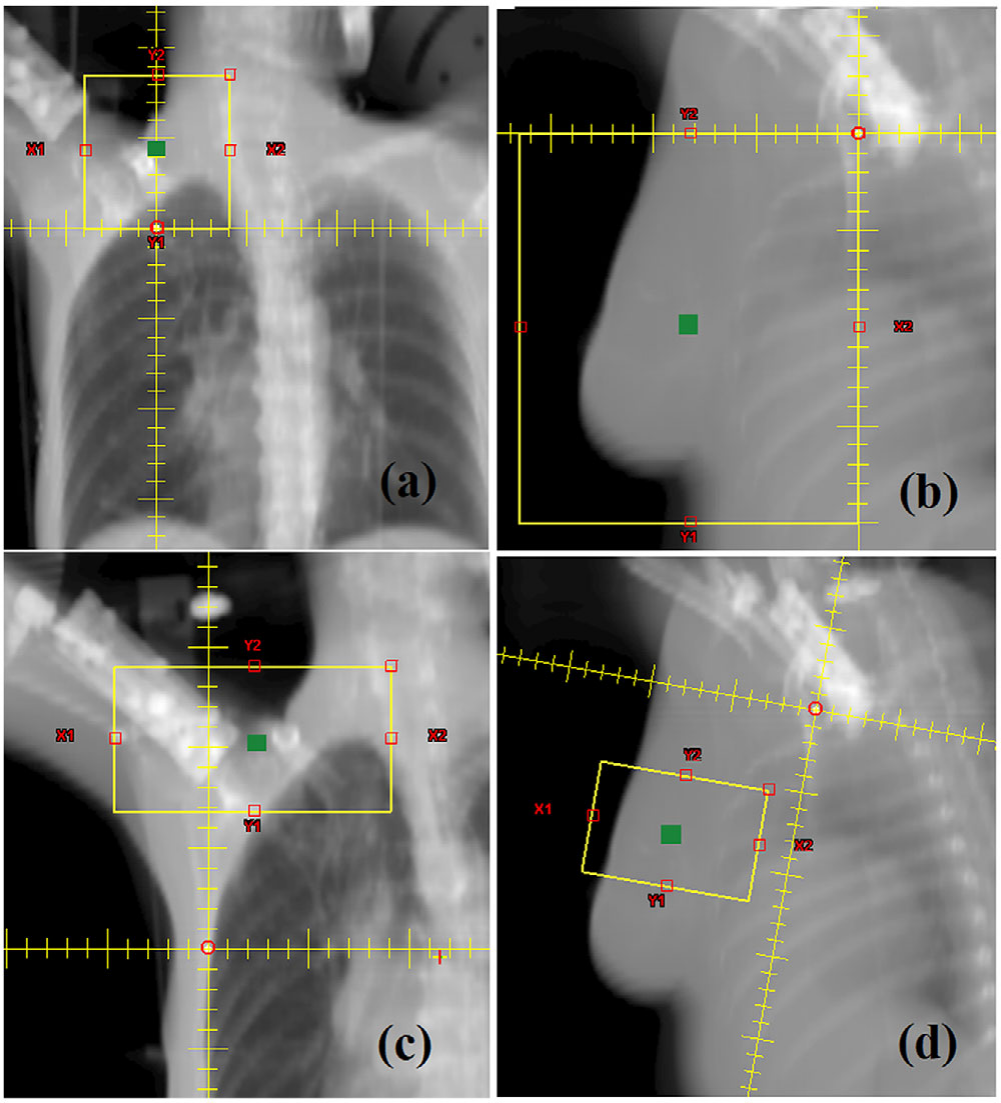
Examples of clinical uses of asymmetric fields are given. (a) Supraclavicular field using a half‐beam. (b) Tangential fields using quarter‐beams. (c) Supraclavicular field using off‐ isocenter beam. (d) Boost field using off‐ isocenter beams. Green marks indicate the EFC. EFC, effective field center.

TFs may be either symmetric or asymmetric, depending on the horizontal distances of the collimator jaws to the isocenter (IC). TFs are usually described using the notation (y_1_,y_2_,x_1_,x_2_), where y_1_, y_2_, x_1_ and x_2_ are the respective collimator jaw positions (in cm) determined at the level of the IC. Asymmetric fields are commonly used in radiotherapy, and regarding treatment planning, there are three special categories of interest (for single‐isocenter techniques), according to the location of the IC with respect to the TF: (a) The IC is centered at an edge of the TF, as in the case of the half‐beams used in the treatment of breast,^4^ as shown in Figure 1a, head and neck,^5^ and craniospinal tumors,^6^, ^7^ (b) The IC is located at a corner of the TF as in the case of a quarter‐beams used for the treatment of the chest wall in breast cancer cases,^4^, ^8^ as shown in Figure 1b, c) The IC is outside TF, in special cases like the off‐isocenter‐beams are used for the treatment of the supraclavicular area and the boost field used for breast tumors,^9^ as shown in Figure 1c and d, respectively.

In all special categories mentioned above, the IC is partially or completely blocked, so MUs are calculated at the effective field center (EFC) rather than the IC. The distance between EFC and IC is called off‐axis distance (*r*). In MU calculations based on the geometric equivalent square field (GESF) concept, the use of dosimetric quantities like S_cp_, PDD, and TMR determined using symmetric fields will lead to significant errors, should the off‐axis ratio (OAR) value of the maximum field size (i.e., 40 × 40 cm^2^) at the location of the EFC is not taken into account. This problem has been studied for square half‐fields (SHF_s_) only, and the errors observed were up to 3% regarding the output factors (S_c_ and S_p_) calculation,^10^ and 7% regarding dose calculations within the phantom/patient.^11^

On the other hand, previous studies have shown that the S_cp_ of an asymmetric field, corrected by the OAR value at the location of EFC, are approximately equal to the corresponding values measured at the IC of their GESF, and in this case differences in MU calculations are less than 1%.^12^, ^13^, ^14^, ^15^, ^16^, ^17^ Thus, several mathematical formulas were developed to improve the accuracy of calculations for asymmetric fields based on the GESF concept,^12^, ^18^, ^19^, ^20^, ^21^ the simplest of which is the area‐perimeter ratio.^22^ According to these formulas, in order to calculate the S_cp_ for asymmetric fields, the S_c_ and S_p_ of their GESF can be used.^3^, ^23^, ^24^, ^25^, ^26^

In the referenced studies this conclusion has been validated for almost all Varian Linacs, such as Clinac 4/100 with 4 MV beam, 600C with 6 MV beam, 2100C with energies 6 and 10 MV, 2100CD with energies 6 and 15 MV, and the Linacs 2100C, 2300C/D, Clinac‐DHX (with energies 6 and 18MV), but no relevant data exist for Varian Clinac IX accelerators, or the 23 MV beam energy. Most important, few studies have measured the S_cp_ of half‐fields, while fewer studies have measured the S_cp_ of the off‐isocenter and quarter‐fields.^11^, ^17^ Moreover, there are no detailed data regarding the S_cp_ measurements.

The aim of this study is to directly measure the S_cp_ of Varian Clinac IX accelerators for the half‐, quarter‐ and off‐isocenter fields and compare it with the values measured at the respective GESFs. This study will be limited to asymmetric square fields (ASFs), for which the effective equivalent field is the same as that of symmetric square field (SSF) whose S_cp_ are tabulated and therefore no approximation is needed. For example, using the notation (y_1_,y_2_,x_1_,x_2_) described above, a SHF with dimensions (0, 10, 5, 5), a square quarter‐field (SQF) with dimensions (0, 10, 0, 10) and a square off‐isocenter field (SOF) with dimensions (−10, 20, 5, 5) have all a respective GESF size of 10 × 10 cm^2^.^21^, ^22^

## 2 MATERIALS AND METHODS

### 2.1 Linear accelerators (LINAC)

This study was performed in two Varian IX Linacs (Varian Medical Systems, Palo Alto, California, USA). The first Linac (L_1_) emits 6 and 23 MV energy photon beams, while the second Linac (L_2_) emits 6 and 18 MV energy photon beams. For each Linac, the dimensions of the treatment field are determined by two pairs of secondary collimators jaws, the upper jaw (y_1_, y_2_) and the lower jaw (x_1_, x_2_). It should be noted that x_1_ and y_1_ can move beyond the IC, for a distance up to −2 and −10 cm, respectively.

**FIGURE 2:**
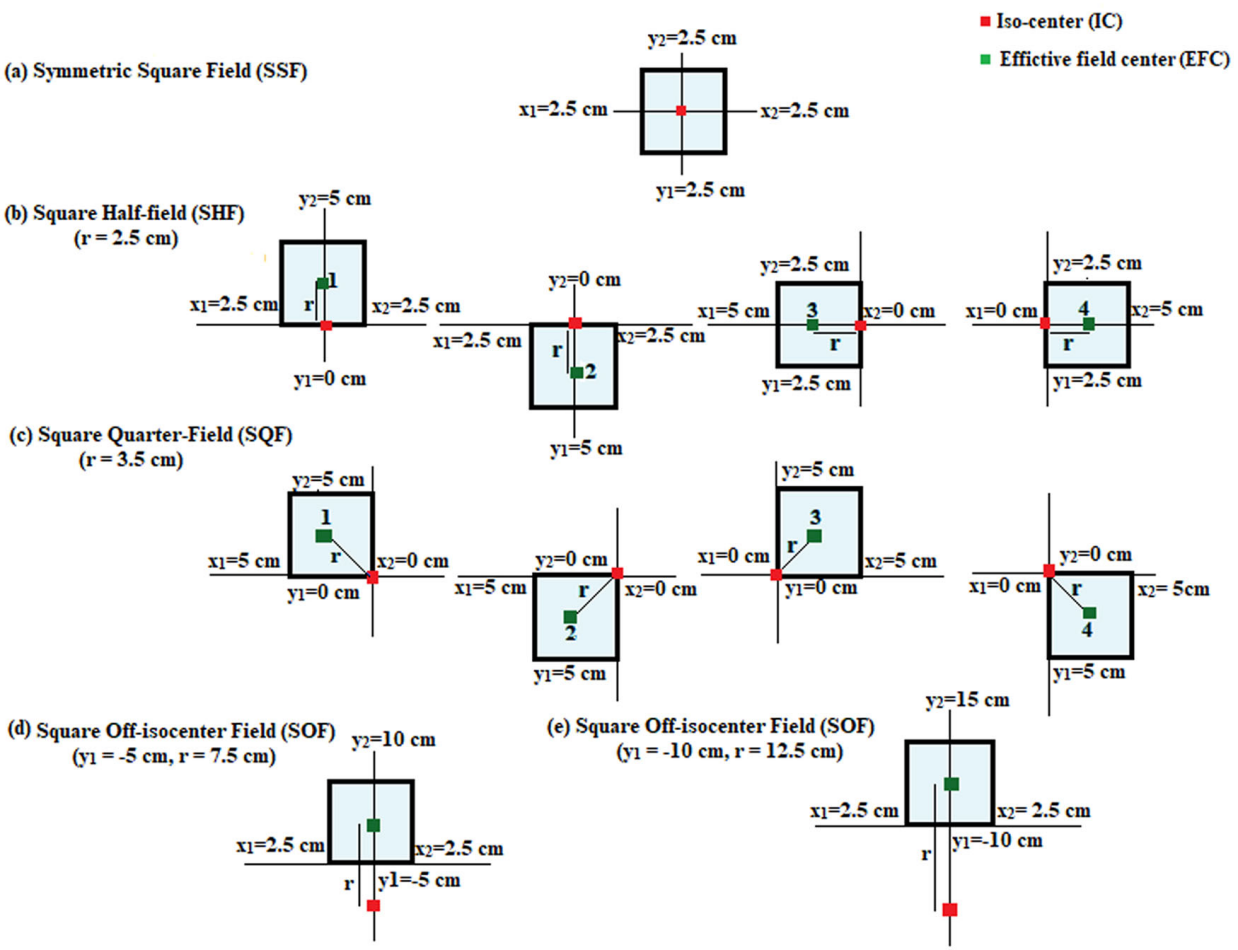
Examples of the geometry of the studied treatment fields are given. Red and green marks indicate the IC and EFC locations, respectively, and *r* is the distance between them. For SHF (b) and SQF (c) there are four different EFC locations (marked with 1, 2, 3 and 4), for the different field size sets. EFC, effective field center; IC, isocenter; SHF, square half‐field.

When y_1_, y_2_ x_1_, x_2_, are all equal, the TF is SSF, as shown in Figure 2a. A half‐field is obtained by setting one of these jaws to position 0, and therefore there are four possible SHF sets (y_1_ = 0, or y_2_ = 0, or x_2_ = 0, or x_1_ = 0), which have their EFC located either on the X‐ or Y‐axis, as shown in Figure 2b. A quarter‐field is obtained by setting one jaw of each pair equal to 0 and there are four possible SQF sets (y_1_ = x_2_ = 0, y_2_ = x_2_ = 0, y_1_ = x_1_ = 0 or y_2_ = x_1_ = 0), which have their EFC_s_ located on the diagonal axes, as shown in Figure 2c. Finally, two SQF sets were studied by setting y_1_ = −5 cm and y_1_ = −10 cm, which have their EFCs located on the positive Y‐axis, as shown in Figures 2d and e, respectively. The distance (*r*) between IC and EFC in quarter‐fields was calculated using Equation (1).

(1) $$r\;=\sqrt{{\left(\frac{x_{i}}{2}\right)}^{2}+{\left(\frac{y_{i}}{2}\right)}^{2}}\;$$

where *i* = 1 and/or 2

### 2.2 Water phantom and ionization chamber description

A MP3 water phantom 600 × 500 × 408 mm^3^ (PTW, Freiburg, Germany) was used, connected with TANDEM dual‐channel electrometer, TBA control unit, and hand pendant. Its proper positioning was fine‐tuned using three laser sources and an inclinometer (Tajima, SLANT 100). To measure the charge, a semi‐flex chamber (PTW 31010, 0.125 cc) connected to a PTW UNIDOS electrometer was used. The chamber was located at a depth of 10 cm within the water phantom and the distance of the x‐ray source focus to the water's surface was adjusted to 90 cm and the gantry angle was kept constant at 0°. For each measurement, the ionization chamber was irradiated to 100 MU and the resulting charge (R) value was recorded by the electrometer.

### 2.3 Scp: Measurements and calculations

S_cp_ for SSFs [S_cp_(IC)]

The ionization chamber was placed at the IC of all SSFs which are the respective GESF of one or more of the ASFs studied, that is, 3 × 3, 5 × 5, 10 × 10, 15 × 15, and 20 × 20 cm^2^, plus the 40 × 40 cm^2^. The S_cp_ (IC) was calculated from Equation (2).

(2) $$S_{cp}\;\left(IC\right)=\frac{R_{IC}\left(X\times Y\right)}{R_{IC}\left(10\times 10\;cm^{2}\right)}\;$$

where R_IC_(X × Y) and R_IC_(10 × 10 cm^2^) are the measured charges R at the IC for the SSFs (X = Y) and the reference field (10 × 10 cm^2^), respectively.

Scp for ASF_s_ [Scp(EFC)]

Using the handheld pendant, the ionization chamber was moved at distance (*r*) to the EFC for all the ASF_s_ studied, which are:

1. SHF_s_: 3 × 3 cm^2^ (*r* = 1.5 cm), 5 × 5 cm^2^ (*r* = 2.5 cm), 10 × 10 cm^2^ (*r* = 5 cm), 15 × 15 cm^2^ (r = 7.5 cm), 20 × 20 cm^2^ (*r* = 10 cm).
2. SQF_s_: 3 × 3 cm^2^ (*r* = 2.12 cm), 5 × 5 cm^2^ (*r* = 3.54 cm), 10 × 10 cm^2^ (*r* = 7.07 cm), 15 × 15 cm^2^ (*r* = 10.61 cm), 20 × 20 cm^2^ (*r* = 14.14 cm).
3. SOF_s_ with y_1_ = −5 cm: 3 × 3 cm^2^ (*r* = 6.5 cm), 5 × 5 cm^2^ (*r* = 7.5 cm), 10 × 10 cm^2^ (*r* = 10 cm), 15 × 15 cm^2^ (*r* = 12.5 cm).
4. SOFs with y_1_ = −10 cm: 3 × 3 cm^2^ (*r* = 11.5 cm), 5 × 5 cm^2^ (*r* = 12. 5 cm), 10 × 10 cm^2^ (*r* = 15 cm).

**FIGURE 3:**
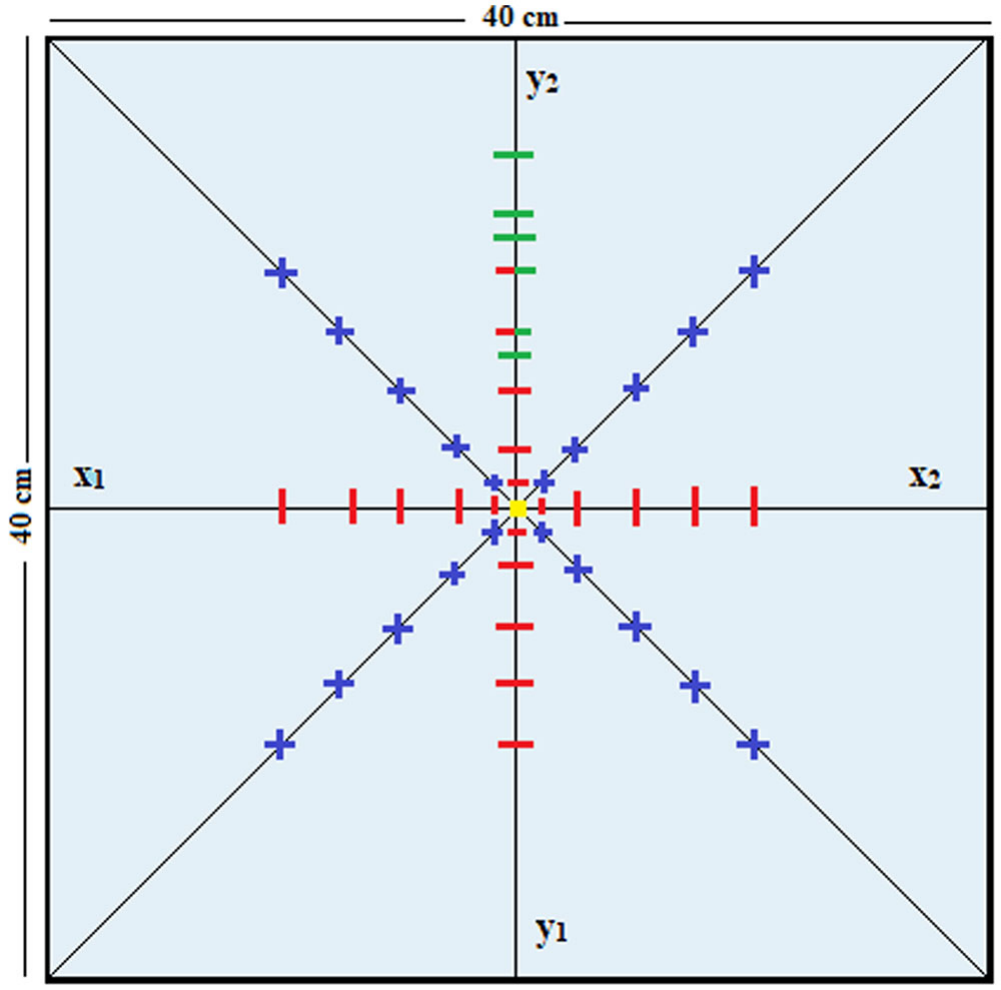
The off‐axis points that correspond to EFC_s_ points of the ASF_s_ used in this study are shown. The red marks on the orthogonal and blue marks on diagonal axes denote the EFC_s_ for the SHFs and SQF_s_, respectively. The green marks on the y_2_ axis denote the EFC_s_ for the SOF_s_. ASFs, asymmetric square fields; EFC, effective field center; SHFs, square half‐fields; SOFs, square off‐isocenter fields; SQFs, square quarter‐fields.

The S_cp_(EFC) was calculated from Equation (3), which is similar to the head scatter (symbolized as H_s_ or S_c_) Equations, defined in previous studies.^12^, ^13^, ^14^

(3) $$S_{cp}\left(\mathrm{EFC}\right)=\frac{R_{EFC}\left(y_{1},y_{2},\;x_{1},\;x_{2},\;r\right)}{R_{IC}\left(10\times 10cm^{2}\right)\times OAR\left(r\right)}$$

where R_EFC_ (y_1_, y_2_, x_1_, x_2_, r) and R_IC_ (10 × 10 cm^2^) are the charges measured at the location of EFC and IC, respectively, and OAR(r) is the OAR measured in a water phantom for a point at a perpendicular distance *r* cm from the central axis for the maximum field size (40 × 40 cm^2^),^27^, ^28^, ^29^, ^30^ as defined in Equation (4).

(4) $$OAR\left(r\right)=\frac{R_{r}\left(40\times 40\;cm^{2}\right)}{R_{IC}\left(40\times 40\;cm^{2}\right)}$$

where R_r_ (40 × 40 cm^2^) is the measured charge at a distance *r* from the IC and R_IC_ (40×40 cm^2^) is the measured charge at the IC for 40 × 40 cm^2^ field size. In Figure 3 are shown the off‐axis points that correspond to the EFCs of the ASFs used in this study. For the measurement of R_r,_ the largest distance (*r*) from the IC was 15 cm, being within 80% of the largest field size and away from the field edges.^28^, ^31^, ^32^

To clarify the order of measurements and calculations made in this study, it must be stressed that before performing any measurements with ASFs, the values of the charge R_IC_(x, y) of all SSFs (which are the GESF of one or more of the ASFs studied, plus the 40 × 40 cm^2^) were first recorded, with the ion chamber positioned at the IC and irradiation of the phantom with 100 MU. For S_cp_(EFC) measurements with ASFs, an example of the measurements performed will be given for the SQF 5 × 5 cm^2^. The collimator jaws were first adjusted at (5, 0, 5, 0) and the chamber was moved at the respective EFC, which is on the diagonal (at distance *r* = 3.54 cm), the phantom was irradiated using 100 MU, and the value of the charge R_EFC_(5, 0, 5, 0, *r* = 3.5 cm) was recorded (to get the numerator of Equation 3). Then, with the chamber kept the same place, the collimator jaws were adjusted to get the maximum SSF 40 × 40 cm^2^, and the reading R_r_(40 × 40 cm^2^) was recorded for an irradiation with 100 MU (to get the numerator of Equation 4). The same procedure was followed for the measurements with all ASF_s_ studied.

For the cases of SHF_s_ and SQF_s_, the S_cp_(EFC) was calculated (using Equations 3 and 4), at four clinically possible locations having the same distance *r* from the IC, as shown in Figures 2b and c, respectively. The final S_cp_(EFC) was defined as the average of the four values as shown in Equation (5). This consideration in calculating S_cp_(EFC) is similar to the diagonal normalized flatness (F_DN_) and OAR calculations defined in previous studies.^31^, ^33^, ^34^

(5) $$\bar{S_{cp}}\left(EFC\right)\;=\frac{\sum_{i=1}^{4}S_{cp}{\left(EFC\right)}_{i}}{4}\;$$

The difference (D_f_%) between S_cp_(IC) and S_cp_(EFC) was calculated using Equation (6).

(6) $$D_{f}\%=\frac{\left[S_{cp}\left(EFC\right)-S_{cp}\left(IC\right)\right]}{S_{cp}\left(IC\right)}\times 100\%$$

For each Linac and ASF, at every EFC point located at a distance *r* to the IC, the measurements of both beam energies were performed, to reduce the uncertainty regarding chamber position.

The uncertainty of the S_cp_ measurements was calculated as the standard deviation (STDEV.P) of the three measurements for the SSF_s_ and SOF_s_ including the respective OAR measurements, and the SD of the four averages of the measurements of the four alternative quadrants for the SHF_s_ and SQF_s_ using Equation (7).

(7) $$\Delta S_{\mathrm{cp}}=\frac{\mathrm{STDEV}.\;P\;[S_{\mathrm{cp}}\left(1\right),\;S_{\mathrm{cp}}\left(2\right),\text{…},\;S_{\mathrm{cp}}\left(N\right)]}{\sqrt{N}}\;$$

where *N* is the number of repeated readings. *N* = 3 when calculating S_cp_ for SSF_s_ and SOF_s_, and *N* = 4 when calculating S_cp_ for SHFs and SQF_s_.

## 3 RESULTS

More than 400 measurements were made in the two Linacs to calculate OAR(r) and then S_cp_ at various points located on one horizontal plane parallel to the water phantom surface at a depth of 10 cm. These points represent the EFCs of the ASF_s_ and the IC of the SSF. All measurements and calculations are listed in Tables 1, 2, 3, 4. These tables display the comparison of the individual S_cp_ values calculated for all ASFs (SHF_s_, SQF_s_, and SOF_s_) with their respective GESF_s_.

**TABLE 1:** Total scatter factors (S_cp_) for symmetric (SSF), half (SHF), quarter (SQF) and off‐isocenter (SOF) fields of 6 MV photon beam generated by Linac 1. *r*(cm): The distance between IC and EFC. OAR(r): Off‐axis ratio. Ratio 1 = R(ASF)/R(SSF). Ratio 2 = S_cp_(ASF)/S_cp_(SSF). The S_cp_, $\bar{S_{\mathrm{cp}}}$, D_f_ %, and $\Delta_{S_{\mathrm{cp}}}$ were calculated from Equations (3), (5), (6), and (7), respectively.

		Jaw setting (cm)												
Field size (cm^2^)	Field shape	y_1_	y_2_	x_1_	x_2_	Area (cm^2^)	*r* (cm)	Charge R (nc)	Ratio 1	OAR(r)	S_cp_	Ratio 2	$\bar{S_{\mathrm{cp}}}\;\pm \Delta S_{cp}$	$\bar{D_{f}}\pm \;\Delta D_{f}\;\%$
**3 × 3**	**SSF**	**1.5**	**1.5**	**1.5**	**1.5**	**9**	**0**	**2.426**	**1.000**	**1**	**0.827**	**1.000**	**0.827 ± 0.001**	**0.0**
	**SHF**	0	3	1.5	1.5	9	1.5	2.438	1.005	1.003	0.828	1.002	0.830 ± 0.001	0.4 ± 0.2
		3	0	1.5	1.5	9	1.5	2.451	1.010	1.007	0.830	1.003		
		1.5	1.5	0	3	9	1.5	2.456	1.012	1.005	0.833	1.007		
		1.5	1.5	3	0	9	1.5	2.435	1.004	1.002	0.828	1.002		
	**SQF**	*3*	*0*	*3*	*0*	*9*	*2.12*	*2.454*	*1.012*	*1.006*	*0.831*	*1.006*	*0.831 ± 0.000*	*0.4 ± 0.1*
		*3*	*0*	*0*	*3*	*9*	*2.12*	*2.467*	*1.017*	*1.012*	*0.831*	*1.005*		
		*0*	*3*	*0*	*3*	*9*	*2.12*	*2.46*	*1.014*	*1.01*	*0.830*	*1.004*		
		*0*	*3*	*3*	*0*	*9*	*2.12*	*2.442*	*1.007*	*1.003*	*0.830*	*1.004*		
	**SOF**	***−5***	***8***	***1.5***	***1.5***	***9***	***6.5***	***2.49***	***1.026***	***1.016***	***0.835***	***1.010***	***0.835 ± 0.001***	***1.0 ± 0.2***
		***−10***	***13***	***1.5***	***1.5***	***9***	***11.5***	***2.488***	***1.017***	***1.01***	***0.840***	***1.015***	***0.840 ± 0.001***	***1.5 ± 0.2***
**5 × 5**	**SSF**	**2.5**	**2.5**	**2.5**	**2.5**	**25**	**0**	**2.615**	**1.000**	**1**	**0.891**	**1.000**	**0.891 ± 0.001**	**0.0**
	**SHF**	0	5	2.5	2.5	25	2.5	2.643	1.011	1.005	0.896	1.006	0.898 ± 0.001	0.8 ± 0.1
		5	0	2.5	2.5	25	2.5	2.661	1.018	1.009	0.899	1.009		
		2.5	2.5	0	5	25	2.5	2.674	1.023	1.013	0.900	1.009		
		2.5	2.5	5	0	25	2.5	2.643	1.011	1.003	0.898	1.008		
	**SQF**	*5*	*0*	*5*	*0*	*25*	*3.54*	*2.669*	*1.021*	*1.009*	*0.902*	*1.012*	*0.901 ± 0.001*	*1.1 ± 0.1*
		*5*	*0*	*0*	*5*	*25*	*3.54*	*2.694*	*1.030*	*1.018*	*0.902*	*1.012*		
		*0*	*5*	*0*	*5*	*25*	*3.54*	*2.677*	*1.024*	*1.014*	*0.900*	*1.010*		
		*0*	*5*	*5*	*0*	*25*	*3.54*	*2.65*	*1.013*	*1.004*	*0.900*	*1.009*		
	**SOF**	***−5***	***10***	***2.5***	***2.5***	***25***	***7.5***	***2.706***	***1.035***	***1.016***	***0.908***	***1.019***	***0.908 ± 0.001***	***1.9 ± 0.1***
		***−10***	***15***	***2.5***	***2.5***	***25***	***12.5***	***2.71***	***1.036***	***1.007***	***0.917***	***1.029***	***0.917 ± 0.001***	***2.9 ± 0.2***
**10 × 10**	**SSF**	**5**	**5**	**5**	**5**	**100**	**0**	**2.934**	**1.000**	**1**	**1.000**	**1.000**	**1.00 ± 0.001**	**0.0**
	**SHF**	0	10	5	5	100	5	2.997	1.021	1.015	1.006	1.006	1.010 ± 0.0.001	1.0 ± 0.2
		10	0	5	5	100	5	3.026	1.031	1.022	1.009	1.009		
		5	5	0	10	100	5	3.042	1.037	1.026	1.011	1.011		
		5	5	10	0	100	5	2.993	1.020	1.007	1.013	1.013		
	**SQF**	*10*	*0*	*10*	*0*	*100*	*7.07*	*3.018*	*1.029*	*1.014*	*1.014*	*1.014*	*1.012 ± 0.002*	*1.2* ± 0.2
		*10*	*0*	*0*	*10*	*100*	*7.07*	*3.052*	*1.040*	*1.033*	*1.007*	*1.007*		
		*0*	*10*	*0*	*10*	*100*	*7.07*	*3.045*	*1.038*	*1.027*	*1.011*	*1.011*		
		*0*	*10*	*10*	*0*	*100*	*7.07*	*3.009*	*1.026*	*1.01*	*1.015*	*1.015*		
	**SOF**	***−5***	***15***	***5***	***5***	***100***	***10***	***3.03***	***1.033***	***1.014***	***1.018***	***1.018***	***1.018 ± 0.001***	***1.8 ± 0.1***
		***−10***	***20***	***5***	***5***	***100***	***15***	***3.048***	***1.039***	***0.994***	***1.045***	***1.045***	***1.045 ± 0.001***	***4.5 ± 0.1***
**15 × 15**	**SSF**	**7.5**	**7.5**	**7.5**	**7.5**	**225**	**0**	**3.12**	**1.000**	**1**	**1.063**	**1.000**	**1.063 ± 0.001**	**0.0**
	**SHF**	0	15	7.5	7.5	225	7.5	3.23	1.035	1.016	1.084	1.019	1.076 ± 0.003	1.2 ± 0.3
		15	0	7.5	7.5	225	7.5	3.208	1.037	1.023	1.069	1.005		
		7.5	7.5	0	15	225	7.5	3.254	1.043	1.034	1.073	1.009		
		7.5	7.5	15	0	225	7.5	3.192	1.023	1.007	1.080	1.016		
	**SQF**	*15*	*0*	*15*	*0*	*225*	*10.6*	*3.221*	*1.032*	*1.01*	*1.087*	*1.022*	*1.082 ± 0.002*	*1.7 ± 0.3*
		*15*	*0*	*0*	*15*	*225*	*10.6*	*3.265*	*1.046*	*1.033*	*1.077*	*1.013*		
		*0*	*15*	*0*	*15*	*225*	*10.6*	*3.253*	*1.043*	*1.028*	*1.079*	*1.014*		
		*0*	*15*	*15*	*0*	*225*	*10.6*	*3.199*	*1.025*	*1.005*	*1.085*	*1.020*		
	**SOF**	***−5***	***20***	***7.5***	***7.5***	***225***	***12.5***	***3.233***	***1.036***	***1.007***	***1.094***	***1.029***	***1.094 ± 0.001***	***2.9 ± 0.1***
**20 × 20**	**SSF**	**10**	**10**	**10**	**10**	**400**	**0**	**3.244**	**1**	**1**	**1.106**	**1.000**	**1.106 ± 0.001**	**0.0**
	**SHF**	0	20	10	10	400	10	3.35	1.033	1.014	1.126	1.018	1.127 ± 0.002	1.9 ± 0.3
		20	0	10	10	400	10	3.369	1.039	1.018	1.128	1.020		
		10	10	0	20	400	10	3.394	1.046	1.033	1.120	1.013		
		10	10	20	0	400	10	3.327	1.026	1.001	1.133	1.025		
	**SQF**	*20*	*0*	*20*	*0*	*400*	*14.1*	*3.361*	*1.036*	*1.001*	*1.144*	*1.035*	*1.138* ± *0.003*	*2.9 ± 0.4*
		*20*	*0*	*0*	*20*	*400*	*14.1*	*3.412*	*1.052*	*1.029*	*1.130*	*1.022*		
		*0*	*20*	*0*	*20*	*400*	*14.1*	*3.401*	*1.048*	*1.023*	*1.133*	*1.025*		
		*0*	*20*	*20*	*0*	*400*	*14.1*	*3.352*	*1.033*	*0.998*	*1.145*	*1.035*		

Abbreviations: ASF, asymmetric square fields; EFC, effective field center; IC, isocenter; SHF, square half‐field; SOF, square off‐isocenter field; SQF, square quarter‐field; SSF, symmetric square field.

**TABLE 2:** Total scatter factors (S_cp_) for symmetric (SSF), half (SHF), quarter (SQF) and off‐isocenter (SOF) fields of 23 MV photon beam generated by Linac 1. *r* (cm): The distance between IC and EFC. OAR(r): Off‐axis ratio. Ratio 1 = R(ASF)/R(SSF). Ratio 2 = S_cp_(ASF)/S_cp_(SSF). The S_cp_, $\bar{S_{\mathrm{cp}}}$, D_f_ %, and $\Delta_{S_{\mathrm{cp}}}$ were calculated from Equations (3), (5), (6), and (7), respectively.

		Jaw setting (cm)												
Field size (cm^2^)	Field shape	y_1_	y_2_	x_1_	x_2_	Area (cm^2^)	*r*(cm)	Charge R (nc)	Ratio 1	OAR(r)	S_cp_	Ratio 2	$\bar{S_{\mathrm{cp}}}\;\pm \Delta S_{cp}$	$\bar{D_{f}}\pm \;\Delta D_{f}\;\%$
**3 × 3**	**SSF**	**1.5**	**1.5**	**1.5**	**1.5**	**9**	**0**	**2.877**	**1.000**	**1**	**0.829**	**1.000**	**0.829 ± 0.001**	**0.0**
	**SHF**	0	3	1.5	1.5	9	1.5	2.867	0.997	0.995	0.830	1.002	0.831 ± 0.001	0.3 ± 0.2
		3	0	1.5	1.5	9	1.5	2.937	1.021	1.015	0.833	1.006		
		1.5	1.5	0	3	9	1.5	2.923	1.016	1.014	0.830	1.002		
		1.5	1.5	3	0	9	1.5	2.887	1.003	1.002	0.830	1.001		
	**SQF**	*3*	*0*	*3*	*0*	*9*	*2.12*	*2.934*	*1.020*	*1.013*	*0.834*	*1.007*	*0.832 ± 0.001*	*0.4 ± 0.2*
		*3*	*0*	*0*	*3*	*9*	*2.12*	*2.966*	*1.031*	*1.025*	*0.833*	*1.006*		
		*0*	*3*	*0*	*3*	*9*	*2.12*	*2.903*	*1.009*	*1.008*	*0.829*	*1.001*		
		*0*	*3*	*3*	*0*	*9*	*2.12*	*2.88*	*1.001*	*0.998*	*0.831*	*1.003*		
	**SOF**	***−5***	***8***	***1.5***	***1.5***	***9***	***6.5***	***2.974***	***1.034***	***1.03***	***0.832***	***1.004***	***0.832 ± 0.001***	***0.4 ± 0.2***
		***−10***	***13***	***1.5***	***1.5***	***9***	***11.5***	***3.024***	***1.051***	***1.038***	***0.839***	***1.013***	***0.839 ± 0.001***	***1.3 ± 0.2***
**5 × 5**	**SSF**	**2.5**	**2.5**	**2.5**	**2.5**	**25**	**0**	**3.186**	**1.000**	**1**	**0.918**	**1.000**	**0.918 ± 0.001**	**0.0**
	**SHF**	0	5	2.5	2.5	25	2.5	3.202	1.005	1.005	0.918	1.000	0.920 ± 0.001	0.3 ± 0.2
		5	0	2.5	2.5	25	2.5	3.299	1.035	1.032	0.921	1.003		
		2.5	2.5	0	5	25	2.5	3.286	1.031	1.027	0.922	1.004		
		2.5	2.5	5	0	25	2.5	3.235	1.015	1.013	0.920	1.002		
	**SQF**	*5*	*0*	*5*	*0*	*25*	*3.54*	*3.304*	*1.037*	*1.028*	*0.926*	*1.009*	*0.925 ± 0.001*	*0.8 ± 0.2*
		*5*	*0*	*0*	*5*	*25*	*3.54*	*3.369*	*1.057*	*1.046*	*0.928*	*1.011*		
		*0*	*5*	*0*	*5*	*25*	*3.54*	*3.291*	*1.033*	*1.026*	*0.924*	*1.007*		
		*0*	*5*	*5*	*0*	*25*	*3.54*	*3.254*	*1.021*	*1.015*	*0.923*	*1.006*		
	**SOF**	***−5***	***10***	***2.5***	***2.5***	***25***	***7.5***	***3.307***	***1.038***	***1.032***	***0.923***	***1.006***	***0.923 ± 0.001***	***0.6 ± 0.1***
		***−10***	***15***	***2.5***	***2.5***	***25***	***12.5***	***3.359***	***1.054***	***1.038***	***0.932***	***1.016***	***0.932 ± 0.001***	***1.6 ± 0.1***
**10 × 10**	**SSF**	**5**	**5**	**5**	**5**	**100**	**0**	**3.472**	**1.000**	**1**	**1.000**	**1.000**	**1.00 ± 0.001**	**0.0**
	**SHF**	0	10	5	5	100	5	3.565	1.027	1.022	1.005	1.005	1.008 ± 0.002	0.8 ± 0.3
		10	0	5	5	100	5	3.648	1.051	1.043	1.007	1.007		
		5	5	0	10	100	5	3.656	1.053	1.048	1.005	1.005		
		5	5	10	0	100	5	3.6	1.037	1.022	1.015	1.015		
	**SQF**	*10*	*0*	*10*	*0*	*100*	*7.07*	*3.633*	*1.046*	*1.035*	*1.011*	*1.011*	*1.009 ± 0.001*	*0.9 ± 0.1*
		*10*	*0*	*0*	*10*	*100*	*7.07*	*3.691*	*1.063*	*1.055*	*1.008*	*1.008*		
		*0*	*10*	*0*	*10*	*100*	*7.07*	*3.637*	*1.048*	*1.039*	*1.008*	*1.008*		
		*0*	*10*	*10*	*0*	*100*	*7.07*	*3.59*	*1.034*	*1.025*	*1.009*	*1.009*		
	**SOF**	***−5***	***15***	***5***	***5***	***100***	***10***	***3.643***	***1.049***	***1.039***	***1.010***	***1.010***	***1.010 ± 0.001***	***1.0 ± 0.1***
		***−10***	***20***	***5***	***5***	***100***	***15***	***3.658***	***1.054***	***1.033***	***1.020***	***1.020***	***1.020 ± 0.001***	***2.0 ± 0.1***
**15 × 15**	**SSF**	**7.5**	**7.5**	**7.5**	**7.5**	**225**	**0**	**3.601**	**1.000**	**1**	**1.037**	**1.000**	**1.037 ± 0.001**	**0.0**
	**SHF**	0	15	7.5	7.5	225	7.5	3.736	1.037	1.032	1.043	1.005	1.044 ± 0.001	0.7 ± 0.1
		15	0	7.5	7.5	225	7.5	3.806	1.057	1.048	1.046	1.009		
		7.5	7.5	0	15	225	7.5	3.812	1.059	1.054	1.042	1.004		
		7.5	7.5	15	0	225	7.5	3.735	1.037	1.029	1.045	1.008		
	**SQF**	*15*	*0*	*15*	*0*	*225*	*10.6*	*3.813*	*1.059*	*1.042*	*1.054*	*1.016*	*1.049 ± 0.002*	*1.2 ± 0.2*
		*15*	*0*	*0*	*15*	*225*	*10.6*	*3.867*	*1.074*	*1.062*	*1.049*	*1.011*		
		*0*	*15*	*0*	*15*	*225*	*10.6*	*3.807*	*1.057*	*1.05*	*1.044*	*1.007*		
		*0*	*15*	*15*	*0*	*225*	*10.6*	*3.769*	*1.047*	*1.033*	*1.051*	*1.013*		
	**SOF**	***−5***	***20***	***7.5***	***7.5***	***225***	***12.5***	***3.797***	***1.054***	***1.038***	***1.054***	***1.016***	***1.054 ± 0.001***	***1.6 ± 0.1***
**20 × 20**	**SSF**	**10**	**10**	**10**	**10**	**400**	**0**	**3.683**	**1**	**1**	**1.061**	**1.000**	**1.061 ± 0.001**	**0.0**
	**SHF**	0	20	10	10	400	10	3.863	1.049	1.039	1.071	1.010	1.073 ± 0.001	1.2 ± 0.1
		20	0	10	10	400	10	3.929	1.067	1.054	1.074	1.012		
		10	10	0	20	400	10	3.945	1.071	1.059	1.073	1.011		
		10	10	20	0	400	10	3.864	1.049	1.035	1.075	1.014		
	**SQF**	*20*	*0*	*20*	*0*	*400*	*14.1*	*3.92*	*1.064*	*1.039*	*1.087*	*1.024*	*1.082 ± 0.002*	*2.0 ± 0.2*
		*20*	*0*	*0*	*20*	*400*	*14.1*	*3.965*	*1.077*	*1.06*	*1.077*	*1.016*		
		*0*	*20*	*0*	*20*	*400*	*14.1*	*3.927*	*1.066*	*1.049*	*1.078*	*1.016*		
		*0*	*20*	*20*	*0*	*400*	*14.1*	*3.885*	*1.055*	*1.032*	*1.084*	*1.022*		

Abbreviations: ASF, asymmetric square fields; EFC, effective field center; IC, isocenter; SHF, square half‐field; SOF, square off‐isocenter field; SQF, square quarter‐field; SSF, symmetric square field.

**TABLE 3:** Total scatter factors (S_cp_) for symmetric (SSF), half (SHF), quarter (SQF) and off‐isocenter (SOF) fields of 6 MV photon beam generated by Linac 2. *r* (cm): The distance between IC and EFC. OAR(r): Off‐axis ratio. Ratio 1 = R(ASF)/R(SSF). Ratio 2 = S_cp_(ASF)/S_cp_(SSF). The S_cp_, $\bar{S_{\mathrm{cp}}}$, D_f_ %, and $\Delta_{S_{\mathrm{cp}}}$ were calculated from Equations (3), (5), (6), and (7), respectively.

		Jaw setting (cm)												
Field size (cm^2^)	Field shape	y_1_	y_2_	x_1_	x_2_	Area (cm^2^)	*r*(cm)	Charge R (nc)	Ratio 1	OAR(r)	S_cp_	Ratio 2	$\bar{S_{\mathrm{cp}}}\;\pm \Delta S_{cp}$	$\bar{D_{f}}\pm \;\Delta D_{f}\;\%$
**3 × 3**	**SSF**	**1.5**	**1.5**	**1.5**	**1.5**	**9**	**0**	**2.5**	**1.000**	**1**	**0.828**	**1.000**	**0.828 ± 0.001**	**0.0**
	**SHF**	0	3	1.5	1.5	9	1.5	2.529	1.012	1.005	0.833	1.007	0.831 ± 0.001	0.4 ± 0.2
		3	0	1.5	1.5	9	1.5	2.529	1.012	1.01	0.829	1.002		
		1.5	1.5	0	3	9	1.5	2.518	1.007	1.003	0.831	1.004		
		1.5	1.5	3	0	9	1.5	2.53	1.012	1.008	0.831	1.004		
	**SQF**	*3*	*0*	*3*	*0*	*9*	*2.12*	*2.551*	*1.020*	*1.01*	*0.836*	*1.010*	*0.835 ± 0.001*	*0.8* ± 0.2
		*3*	*0*	*0*	*3*	*9*	*2.12*	*2.529*	*1.012*	*1.008*	*0.831*	*1.004*		
		*0*	*3*	*0*	*3*	*9*	*2.12*	*2.538*	*1.015*	*1.004*	*0.837*	*1.011*		
		*0*	*3*	*3*	*0*	*9*	*2.12*	*2.526*	*1.010*	*1.003*	*0.834*	*1.007*		
	**SOF**	***−5***	***8***	***1.5***	***1.5***	***9***	***6.5***	***2.58***	***1.032***	***1.017***	***0.840***	***1.015***	***0.840 ± 0.001***	***1.5*** ± 0.2
		***−10***	***13***	***1.5***	***1.5***	***9***	***11.5***	***2.578***	***1.031***	***1.013***	***0.843***	***1.018***	***0.843 ± 0.001***	***1.8 ± 0.2***
**5 × 5**	**SSF**	**2.5**	**2.5**	**2.5**	**2.5**	**25**	**0**	**2.694**	**1.000**	**1**	**0.892**	**1.000**	**0.892 ± 0.001**	**0.0**
	**SHF**	0	5	2.5	2.5	25	2.5	2.726	1.012	1.006	0.897	1.006	0.897 ± 0.001	0.5 ± 0.2
		5	0	2.5	2.5	25	2.5	2.739	1.017	1.014	0.894	1.003		
		2.5	2.5	0	5	25	2.5	2.722	1.010	1.005	0.897	1.005		
		2.5	2.5	5	0	25	2.5	2.745	1.019	1.012	0.898	1.007		
	**SQF**	*5*	*0*	*5*	*0*	*25*	*3.54*	*2.755*	*1.023*	*1.016*	*0.898*	*1.007*	*0.898 ± 0.001*	*0.7* ± 0.2
		*5*	*0*	*0*	*5*	*25*	*3.54*	*2.74*	*1.017*	*1.011*	*0.897*	*1.006*		
		*0*	*5*	*0*	*5*	*25*	*3.54*	*2.747*	*1.020*	*1.01*	*0.90 1*	*1.010*		
		*0*	*5*	*5*	*0*	*25*	*3.54*	*2.73*	*1.013*	*1.009*	*0.896*	*1.004*		
	**SOF**	***−5***	***10***	***2.5***	***2.5***	***25***	***7.5***	***2.788***	***1.035***	***1.017***	***0.908***	***1.018***	***0.908 ± 0.001***	***1.8 ± 0.1***
		***−10***	***15***	***2.5***	***2.5***	***25***	***12.5***	***2.794***	***1.037***	***1.009***	***0.917***	***1.028***	***0.917 ± 0.001***	***2.8 ± 0.2***
**10 × 10**	**SSF**	**5**	**5**	**5**	**5**	**100**	**0**	**3.02**	**1.000**	**1**	**1.000**	**1.000**	**1.00 ± 0.001**	**0.0**
	**SHF**	0	10	5	5	100	5	3.087	1.022	1.015	1.007	1.007	1.008 ± 0.000	0.8 ± 0.1
		10	0	5	5	100	5	3.111	1.030	1.022	1.008	1.008		
		5	5	0	10	100	5	3.086	1.022	1.015	1.007	1.007		
		5	5	10	0	100	5	3.118	1.032	1.023	1.009	1.009		
	**SQF**	*10*	*0*	*10*	*0*	*100*	*7.07*	*3.141*	*1.040*	*1.026*	*1.014*	*1.014*	*1.010 ± 0.001*	*1.0 ± 0.2*
		*10*	*0*	*0*	*10*	*100*	*7.07*	*3.115*	*1.031*	*1.023*	*1.008*	*1.008*		
		*0*	*10*	*0*	*10*	*100*	*7.07*	*3.098*	*1.026*	*1.016*	*1.010*	*1.010*		
		*0*	*10*	*10*	*0*	*100*	*7.07*	*3.108*	*1.029*	*1.02*	*1.009*	*1.009*		
	**SOF**	***−5***	***15***	***5***	***5***	***100***	***10***	***3.119***	***1.033***	***1.016***	***1.017***	***1.017***	***1.017 ± 0.001***	***1.7 ± 0.1***
		***−10***	***20***	***5***	***5***	***100***	***15***	***3.138***	***1.039***	***0.998***	***1.041***	***1.041***	***1.041 ± 0.001***	***4.1 ± 0.1***
**15 × 15**	**SSF**	**7.5**	**7.5**	**7.5**	**7.5**	**225**	**0**	**3.216**	**1.000**	**1**	**1.065**	**1.000**	**1.065 ± 0.001**	**0.0**
	**SHF**	0	15	7.5	7.5	225	7.5	3.327	1.035	1.017	1.083	1.017	1.076 ± 0.003	1.1 ± 0.3
		15	0	7.5	7.5	225	7.5	3.305	1.028	1.022	1.071	1.006		
		7.5	7.5	0	15	225	7.5	3.303	1.027	1.021	1.071	1.006		
		7.5	7.5	15	0	225	7.5	3.338	1.038	1.024	1.079	1.014		
	**SQF**	*15*	*0*	*15*	*0*	*225*	*10.6*	*3.351*	*1.042*	*1.022*	*1.086*	*1.020*	*1.083 ± 0.002*	*1.7 ± 0.3*
		*15*	*0*	*0*	*15*	*225*	*10.6*	*3.327*	*1.035*	*1.023*	*1.077*	*1.011*		
		*0*	*15*	*0*	*15*	*225*	*10.6*	*3.342*	*1.039*	*1.016*	*1.089*	*1.023*		
		*0*	*15*	*15*	*0*	*225*	*10.6*	*3.317*	*1.031*	*1.016*	*1.081*	*1.015*		
	**SOF**	***−5***	***20***	***7.5***	***7.5***	***225***	***12.5***	***3.335***	***1.037***	***1.009***	***1.094***	***1.028***	***1.094 ± 0.001***	***2.8 ± 0.1***
**20 × 20**	**SSF**	**10**	**10**	**10**	**10**	**400**	**0**	**3.345**	**1**	**1**	**1.108**	**1.000**	**1.108 ± 0.001**	**0.0**
	**SHF**	0	20	10	10	400	10	3.453	1.032	1.016	1.125	1.016	1.125 ± 0.003	1.6 ± 0.3
		20	0	10	10	400	10	3.475	1.039	1.025	1.123	1.014		
		10	10	0	20	400	10	3.451	1.032	1.02	1.120	1.011		
		10	10	20	0	400	10	3.485	1.042	1.018	1.134	1.023		
	**SQF**	*20*	*0*	*20*	*0*	*400*	*14.1*	*3.504*	*1.048*	*1.013*	*1.145*	*1.034*	*1.140 ± 0.003*	*2.9 ± 0.3*
		*20*	*0*	*0*	*20*	*400*	*14.1*	*3.481*	*1.041*	*1.019*	*1.131*	*1.021*
		*0*	*20*	*0*	*20*	*400*	*14.1*	*3.496*	*1.045*	*1.013*	*1.143*	*1.032*		
		*0*	*20*	*20*	*0*	*400*	*14.1*	*3.475*	*1.039*	*1.009*	*1.140*	*1.030*		

Abbreviations: ASF, asymmetric square fields; EFC, effective field center; IC, isocenter; SHF, square half‐field; SOF, square off‐isocenter field; SQF, square quarter‐field; SSF, symmetric square field.

**TABLE 4:** Total scatter factors (S_cp_) for symmetric (SSF), half (SHF), quarter (SQF) and off‐isocenter (SOF) fields of 18MV photon beam generated by Linac 2. *r* (cm): The distance between IC and EFC. OAR(r): Off‐axis ratio. Ratio 1 = R(ASF)/R(SSF). Ratio 2 = S_cp_(ASF)/S_cp_(SSF). The S_cp_, $\bar{S_{\mathrm{cp}}}$, D_f_ %, and $\Delta_{S_{\mathrm{cp}}}$ were calculated from Equations (3), (5), (6), and (7), respectively.

		Jaw setting (cm)												
Field size (cm^2^)	Field shape	y_1_	y_2_	x_1_	x_2_	Area (cm^2^)	*r*(cm)	Charge R (nc)	Ratio 1	OAR(r)	S_cp_	Ratio 2	$\bar{S_{\mathrm{cp}}}\;\pm \Delta S_{cp}$	$\bar{D_{f}}\pm \;\Delta D_{f}\;\%$
**3 × 3**	**SSF**	**1.5**	**1.5**	**1.5**	**1.5**	**9**	**0**	**2.937**	**1.000**	**1**	**0.832**	**1.000**	**0.832 ± 0.001**	**0.0**
	**SHF**	0	3	1.5	1.5	9	1.5	2.98	1.015	1.005	0.840	1.010	0.838 ± 0.001	0.6 ± 0.2
		3	0	1.5	1.5	9	1.5	2.964	1.009	1.003	0.838	1.006		
		1.5	1.5	0	3	9	1.5	2.928	0.997	0.996	0.833	1.001		
		1.5	1.5	3	0	9	1.5	2.995	1.020	1.012	0.839	1.008		
	**SQF**	*3*	*0*	*3*	*0*	*9*	*2.12*	*3.001*	*1.022*	*1.013*	*0.840*	*1.009*	*0.839 ± 0.001*	*0.8 ± 0.2*
		*3*	*0*	*0*	*3*	*9*	*2.12*	*2.948*	*1.004*	*1*	*0.836*	*1.004*		
		*0*	*3*	*0*	*3*	*9*	*2.12*	*2.971*	*1.012*	*1.002*	*0.840*	*1.010*		
		*0*	*3*	*3*	*0*	*9*	*2.12*	*3.02*	*1.028*	*1.019*	*0.840*	*1.009*		
	**SOF**	***−5***	***8***	***1.5***	***1.5***	***9***	***6.5***	***3.055***	***1.040***	***1.03***	***0.841***	***1.010***	***0.841 ± 0.001***	***1.0 ± 0.2***
		***−10***	***13***	***1.5***	***1.5***	***9***	***11.5***	***3.109***	***1.059***	***1.04***	***0.847***	***1.018***	***0.847 ± 0.001***	***1.8 ± 0.2***
**5 × 5**	**SSF**	**2.5**	**2.5**	**2.5**	**2.5**	**25**	**0**	**3.233**	**1.000**	**1**	**0.916**	**1.000**	**0.916 ± 0.001**	**0.0**
	**SHF**	0	5	2.5	2.5	25	2.5	3.308	1.023	1.015	0.924	1.008	0.922 ± 0.001	0.7 ± 0.2
		5	0	2.5	2.5	25	2.5	3.285	1.016	1.011	0.921	1.005		
		2.5	2.5	0	5	25	2.5	3.263	1.009	1.001	0.924	1.008		
		2.5	2.5	5	0	25	2.5	3.327	1.029	1.024	0.921	1.005		
	**SQF**	*5*	*0*	*5*	*0*	*25*	*3.54*	*3.36*	*1.039*	*1.03*	*0.925*	*1.009*	*0.924 ± 0.001*	*0.9 ± 0.2*
		*5*	*0*	*0*	*5*	*25*	*3.54*	*3.309*	*1.024*	*1.017*	*0.922*	*1.006*		
		*0*	*5*	*0*	*5*	*25*	*3.54*	*3.317*	*1.026*	*1.018*	*0.924*	*1.008*		
		*0*	*5*	*5*	*0*	*25*	*3.54*	*3.375*	*1.044*	*1.032*	*0.927*	*1.012*		
	**SOF**	***−5***	***10***	***2.5***	***2.5***	***25***	***7.5***	***3.37***	***1.042***	***1.033***	***0.925***	***1.009***	***0.925 ± 0.001***	***0.9 ± 0.1***
		***−10***	***15***	***2.5***	***2.5***	***25***	***12.5***	***3.416***	***1.057***	***1.038***	***0.933***	***1.018***	***0.933 ± 0.001***	***1.8 ± 0.1***
**10 × 10**	**SSF**	**5**	**5**	**5**	**5**	**100**	**0**	**3.528**	**1.000**	**1**	**1.000**	**1.000**	**1.00 ± 0.001**	**0.0**
	**SHF**	0	10	5	5	100	5	3.645	1.033	1.027	1.006	1.006	1.006 ± 0.001	0.6 ± 0.2
		10	0	5	5	100	5	3.64	1.032	1.026	1.006	1.006		
		5	5	0	10	100	5	3.607	1.022	1.02	1.002	1.002		
		5	5	10	0	100	5	3.685	1.045	1.036	1.008	1.008		
	**SQF**	*10*	*0*	*10*	*0*	*100*	*7.07*	*3.692*	*1.046*	*1.038*	*1.008*	*1.008*	*1.008 ± 0.001*	*0.8 ± 0.2*
		*10*	*0*	*0*	*10*	*100*	*7.07*	*3.647*	*1.034*	*1.028*	*1.006*	*1.006*		
		*0*	*10*	*0*	*10*	*100*	*7.07*	*3.652*	*1.035*	*1.028*	*1.007*	*1.007*		
		*0*	*10*	*10*	*0*	*100*	*7.07*	*3.7*	*1.049*	*1.038*	*1.010*	*1.010*		
	**SOF**	***−5***	***15***	***5***	***5***	***100***	***10***	***3.713***	***1.052***	***1.042***	***1.010***	***1.010***	***1.010 ± 0.001***	***1.0 ± 0.1***
		***−10***	***20***	***5***	***5***	***100***	***15***	***3.726***	***1.056***	***1.034***	***1.021***	***1.021***	***1.021 ± 0.001***	***2.1 ± 0.1***
**15 × 15**	**SSF**	**7.5**	**7.5**	**7.5**	**7.5**	**225**	**0**	**3.655**	**1.000**	**1**	**1.036**	**1.000**	**1.036 ± 0.001**	**0.0**
	**SHF**	0	15	7.5	7.5	225	7.5	3.809	1.042	1.033	1.045	1.009	1.045 ± 0.001	0.9 ± 0.1
		15	0	7.5	7.5	225	7.5	3.811	1.043	1.032	1.047	1.010		
		7.5	7.5	0	15	225	7.5	3.778	1.034	1.027	1.043	1.006		
		7.5	7.5	15	0	225	7.5	3.846	1.052	1.041	1.047	1.011		
	**SQF**	*15*	*0*	*15*	*0*	*225*	*10.6*	*3.875*	*1.060*	*1.044*	*1.052*	*1.016*	*1.049 ± 0.001*	*1.3* ± 0.2
		*15*	*0*	*0*	*15*	*225*	*10.6*	*3.826*	*1.047*	*1.038*	*1.045*	*1.008*		
		*0*	*15*	*0*	*15*	*225*	*10.6*	*3.841*	*1.051*	*1.039*	*1.048*	*1.011*		
		*0*	*15*	*15*	*0*	*225*	*10.6*	*3.88*	*1.062*	*1.046*	*1.051*	*1.015*		
	**SOF**	***−5***	***20***	***7.5***	***7.5***	***225***	***12.5***	***3.861***	***1.056***	***1.038***	***1.054***	***1.018***	***1.054 ± 0.001***	***1.8*** ± 0.1
**20 × 20**	**SSF**	**10**	**10**	**10**	**10**	**400**	**0**	**3.737**	**1**	**1**	**1.059**	**1.000**	**1.059 ± 0.001**	**0.00**
	**SHF**	0	20	10	10	400	10	3.933	1.052	1.042	1.070	1.010	1.074 ± 0.001	1.4 ± 0.2
		20	0	10	10	400	10	3.941	1.055	1.037	1.077	1.017		
		10	10	0	20	400	10	3.905	1.045	1.033	1.071	1.012		
		10	10	20	0	400	10	3.965	1.061	1.045	1.075	1.015		
	**SQF**	*20*	*0*	*20*	*0*	*400*	*14.1*	*3.988*	*1.067*	*1.041*	*1.086*	*1.025*	*1.082 ± 0.002*	*2.2 ± 0.2*
		*20*	*0*	*0*	*20*	*400*	*14.1*	*3.951*	*1.057*	*1.039*	*1.078*	*1.018*		
		*0*	*20*	*0*	*20*	*400*	*14.1*	*3.958*	*1.059*	*1.039*	*1.080*	*1.019*		
		*0*	*20*	*20*	*0*	*400*	*14.1*	*3.997*	*1.070*	*1.044*	*1.085*	*1.024*		

Abbreviations: ASF, asymmetric square fields; EFC, effective field center; IC, isocenter; SHF, square half‐field; SOF, square off‐isocenter field; SQF, square quarter‐field; SSF, symmetric square field.

**FIGURE 4:**
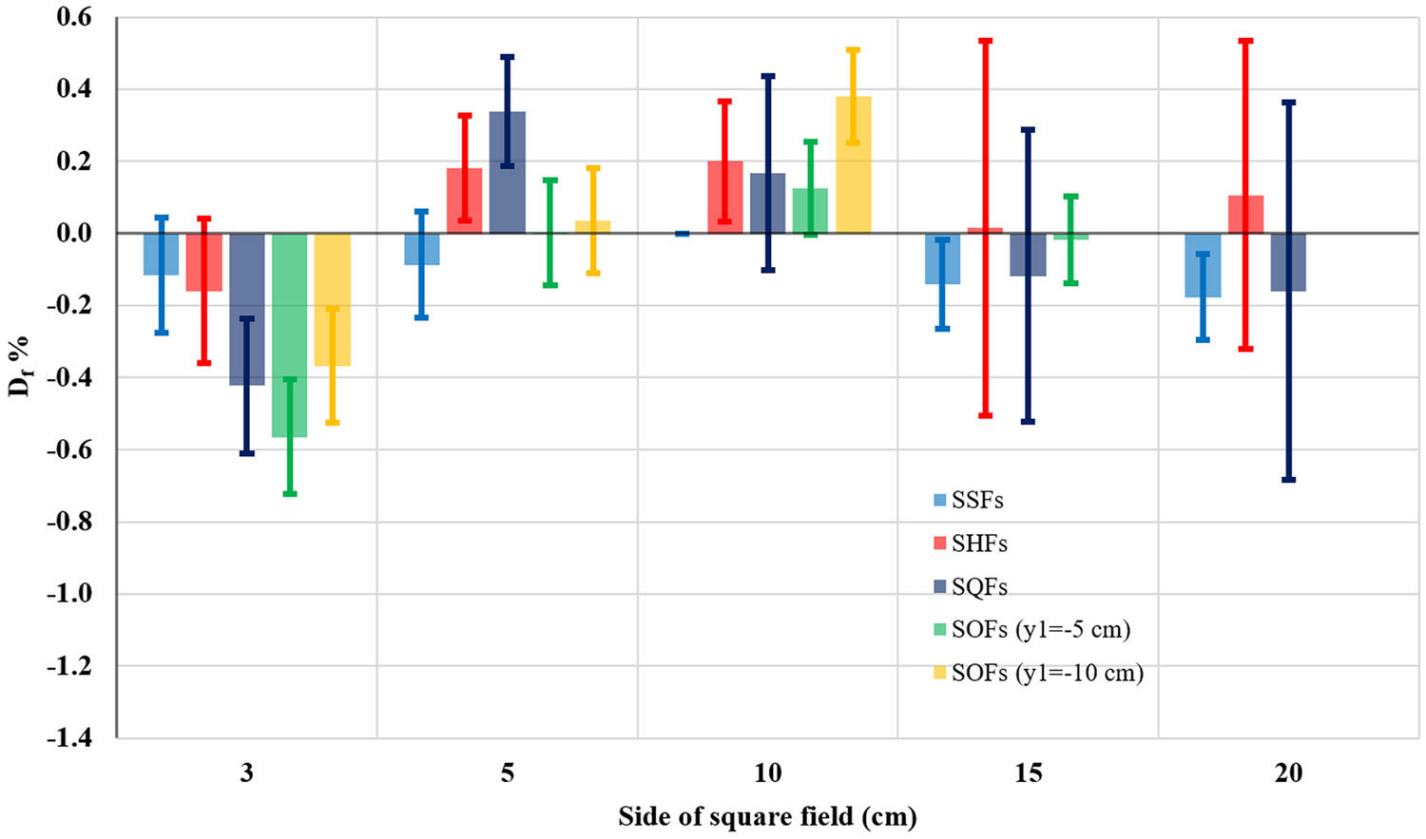
S_cp_ differences D_f_ % between the L_1_ and the L_2_ for the 6 MV energy for all studied fields. S_cp,_ scatter factor.

**FIGURE 5:**
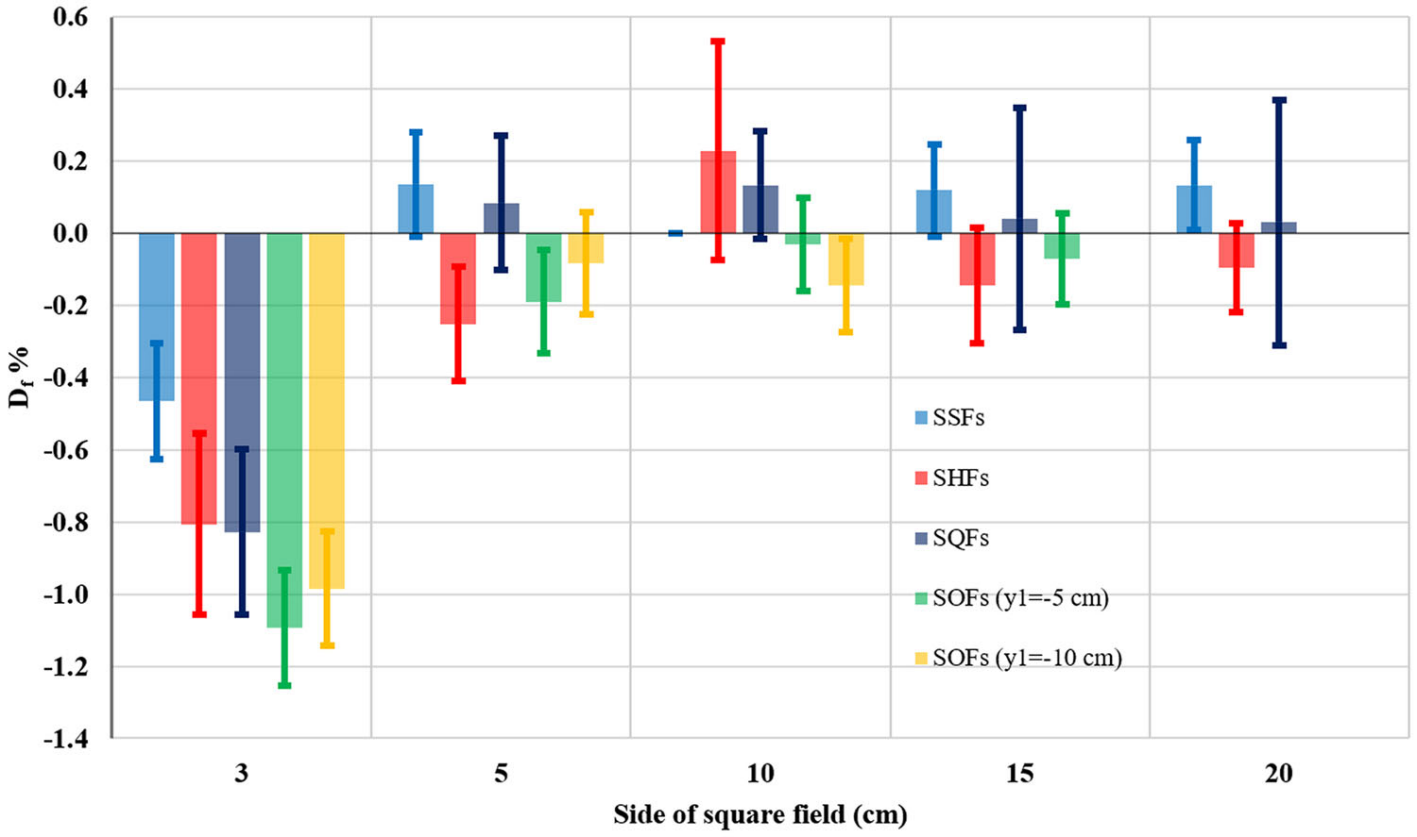
S_cp_ difference D_f_ % between the beam energies of 18 MV (L_1_) and 23 MV (L_2_), for all studied fields. S_cp,_ scatter factor.

Based on these results, there are no significant differences in S_cp_ values calculated for two 6 MV energies for L_1_ and L_2_, as illustrated in Figure 4. The same was true for 18 and 23 MV, except probably for the field 3 × 3 cm^2^ off‐isocenter fields, where the difference was about 1%, as shown in Figure 5. This confirms the accuracy of the calculation methodology followed and its compatibility for both accelerators, so that the average S_cp_ values can be adopted for the two 6 MV beams and respectively for the 18/23 MV x‐ray beam energies.

**FIGURE 6:**
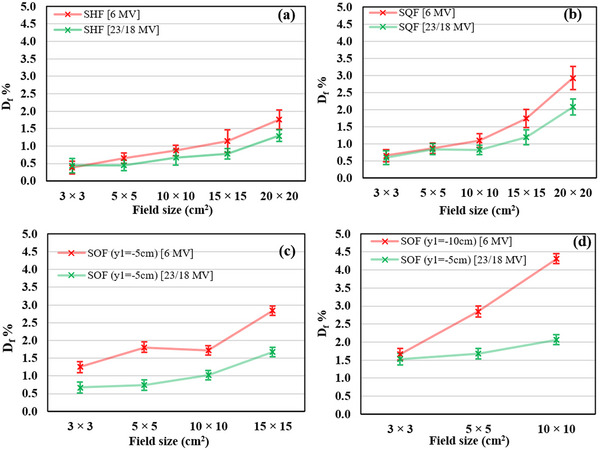
The average S_cp_ differences (D_f_%) between ASF_s_ and GESF for the 6 MV and 18/23 MV photon beam energies. (a) SHF. (b) SQF. (c) and (d) SOF with y_1_ = −5 and y_1_ = −10 cm, respectively. ASFs, asymmetric square fields; GESF, geometric equivalent square field; S_cp,_ scatter factor; SHF, square half‐field; SOF, square off‐isocenter field; SQF, square quarter‐field.

Tables 1, 2, 3, 4 show that the uncertainty associated with the S_cp_ calculation is small with maximum error being 0.3% and 0.2% for energies 6 and 18/23 MV, respectively. It was noted from Figures 4 and 5 that the maximum uncertainty when studying the difference in the S_cp_ between the two accelerators (considering respective TF comparisons) reached 0.5% and 0.3% for 6 and 18/23 MV, respectively. On the other hand, it was found that the estimated uncertainty in calculating the difference in S_cp_ between ASF_s_ and SSF_s_ is less than 1% and ranges from 0.1% to 0.3% and from 0.1% to 0.2%, as shown in Table 5 and Figure 6, for energies 6 and 18/23 MV respectively.

**TABLE 5:** The S_cp_ differences between ASFs (SHFs, SHFs and SOFs) and their equivalent fields SSF_s_ or GESF_s_ for the 6 and 18/23 MV photon energies. $\bar{D_{f}}\%:$ Average D_f_ % values for every two equal energies from Tables 1, 2, 3, 4. The superscripts in parenthesis for the 10×10 fields (6 MV) indicate the distance *r* between the IC and the EFC. With bold are denoted differences from 1% to 2% and with bold italics differences above 2%.

	$\bar{D_{f}}\pm \Delta D_{f}$%: ASFs & SSFs (GESF)for 6 MV				$\bar{D_{f}}\pm \Delta D_{f}$%: ASFs & SSFs (GESF) for 18/23 MV			
			SOFs				SOFs	
Field size (cm^2^)	SHF_s_	SQF_s_	−5 cm	−10 cm	SHF_s_	SQF_s_	−5 cm	−10 cm
3 × 3	0.4 ± 0.2	0.7 ± 0.2	**1.2** ± **0.2**	**1.7** ± 0.2	0.4 ± 02	0.6 ± 0.2	0.7 ± 0.2	**1.5** ± 0.2
5 × 5	0.7 ± 0.1	0.9 ± 0.2	**1.8** ± **0.1**	***2.8*** ± ***0.2***	0.4 ± 0.2	0.8 ± 0.2	0.7 ± 0.1	**1.7** ± **0.1**
10 × 10	0.9^(5 cm)^ ± 0.2	**1.1** ^**(7.1 cm)**^ ± **0.2**	**1.7** ^**(10 cm)**^ ± **0.1**	***4.3*** ^***(15 cm)***^ ± ***0.1***	0.7 ± 0.2	0.8 ± 0.1	1.0 ± 0.1	***2.1*** ± ***0.1***
15 × 15	**1.1 ± 0.3**	**1.7** ± **0.3**	***2.8*** ± ***0.1***		0.8 ± 0.1	**1.2** ± **0.2**	**1.7** ± **0.1**	
20 × 20	**1.8 ± 0.3**	***2.9 ± 0.3***			**1.3** ± **0.2**	***2.1 ± 0.2***		

Abbreviations: ASF, asymmetric square fields; EFC, effective field center; GESF, geometric equivalent square field; IC, isocenter; SHF, square half‐field; SOF, square off‐isocenter field; SQF, square quarter‐field; SSF, symmetric square field.

Table 5 shows the S_cp_ differences between ASF_s_ and their GESF_s_ for the 6 and 18/23 MV photon energies, which are also represented graphically in Figure 6. From Table 5 and Figure 6 it becomes evident that the S_cp_ differences between ASF and their respective GESF, increase gradually with the increasing field size (i.e., with increasing Table's 5 column number). Also, the more asymmetric the field is (SHF → SQF → SOF) the larger the more the increase is (i.e., with increasing Table's 5 row number), as the more asymmetric the field is the larger the distance *r* of its geometric center (EFC) to the IC is. However, these differences reduce with increasing beam energy. Consequently, there are two factors that may affect the increase of S_cp_(EFC), and it can be either both or one of them. The first factor is the method of the jaw setting (SHF, SQF or SOF) and the second factor is the off‐axis distance (*r*).

**FIGURE 7:**
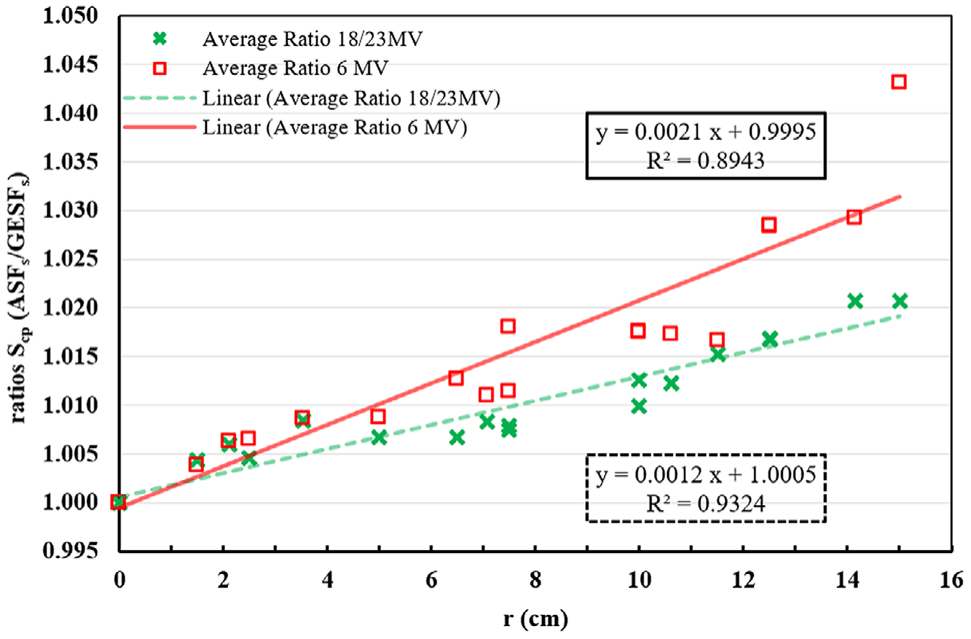
Average S_cp_ difference between ASF_s_ and GESF_s_ for the 6 and 18/23 MV energies. ASFs, asymmetric square fields; GESF, geometric equivalent square field; S_cp,_ scatter factor.

The effect of the method of jaw setting can be investigated by comparing directly the ratio of the measured charge values at the center of two different ASF shapes (e.g., the SOF with dimensions 15 × 15 cm^2^ and 5 × 5 cm^2^) which have the same EFC (e.g., $\frac{R(15\times 15cm^{2},\;r\;=\;12.5cm)}{R(5\times 5cm^{2},\;r\;=\;12.5cm)}$) with the measured charge ratio of their GESF_s_ in the IC (e.g.,$\;\frac{R(15\times 15cm^{2},\;r\;=\;0cm)}{R(5\times 5cm^{2},\;r\;=\;0cm)}$). Table 6 shows that the two ratios are almost equal at all studied energies and the difference between them is less than 0.05%. Since the OAR(r) used to correct the charge measurements is the same for all ASFs having the EFC at same distance *r*, the S_cp_(EFC) is independent of the asymmetric field size or the location of the field margin from the IC for these studied cases. Therefore, this implies that the main factor contributing to the S_cp_ increase is the off‐axis distance (*r*) of the EFC. Indeed, in Figure 7 are shown the S_cp_ differences between ASF_s_ and GESF_s_ (expressed as their ratio) for the 6 and 18/23 MV with relation to the EFC's *r* values. This figure shows that the average S_cp_ ratios increase gradually with *r* and that the average values of 6 MV data, are in almost all cases larger than the respective 18/23 MV data. For 18/23 MV, the differences are within or close to 2% but for 6 MV, the differences exceed 4%.

**TABLE 6:** The field‐size effect test on S_cp_ (EFC). The results were extracted from Tables 1, 2, 3, 4.

	Asymmetric jaw setting (cm)					Ratio (Asym1/Asym2; at same EFC and different shape): Ratio (Sym1. GESF/Sym2.GESF; at IC) [D_f_ %]			
Field size (cm^2^)	y_1_	y_2_	x_1_	x_2_	*r*	6 MV (L_1_)	6 MV (L_2_)	23 MV (L_1_)	18 MV (L_2_)
20 × 20	0	20	10	10	10	1.1056:1.1057	1.1071:1.1076	1.0604:1.0608	1.0593:1.0592
10 × 10	**−**5	15	5	5	10	[−0.0043]	[−0.0479]	[−0.0360]	[0.0010]
15 × 15	0	15	7.5	7.5	7.5	1.1936:1.1931	1.1933:1.1938	1.1297:1.1303	1.1303:1.1305
5 × 5	**−**5	10	2.5	2.5	7.5	[0.0442]	[−0.0365]	[−0.0471]	[−0.0232]
15 × 15	**−**5	20	7.5	7.5	12.5	1.1930:1.1931	1.1936:1.1938	1.1304:1.1303	1.1303:1.1305
5 × 5	**−**10	15	2.5	2.5	12.5	[−0.0107]	[−0.0113]	[0.0123]	[−0.0230]

Abbreviations: ASF, asymmetric square fields; EFC, effective field center; GESF, geometric equivalent square field; IC, isocenter; SHF, square half‐field; SOF, square off‐isocenter field; SQF, square quarter‐field; SSF, symmetric square field.

## 4 DISCUSSION

**FIGURE 8:**
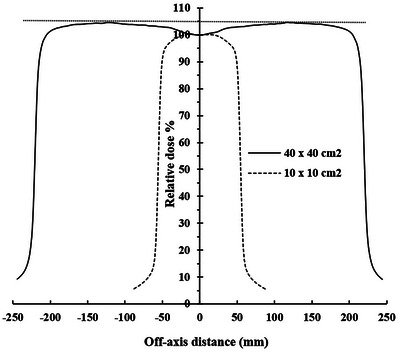
OAR(r) profiles at d_max_ for 23 MV. OAR, off‐axis ratio.

The flattening filter (FF) has a Gaussian‐like shape, and as a result the beam energy of the central ray passing through IC is relatively larger than the rays away from the IC, and thus more penetrating. As a result, OAR(r) profiles at small depths are not flat around the central axis and present their maximum values at a distance from the IC, depending on the field size (as shown in Figure 8).^16^ Daryoush et al.^35^ confirmed that the 18 MV energy photons scatter preferentially in the forward direction, while the 6 MV energy photons scatter in the forward and sides direction with a relative predominance in the forward direction of 0.5%. On the other hand, the S_cp_ increases with decreasing energy for larger field size due to the increasing phantom scatter contribution (S_p_). In asymmetric fields, the EFC shifts to the sides and thus the lateral scatter increases with increasing *r* due to decreasing energy,^16^ as long as the measurement is performed away from the beam edges. This agrees with the results shown in Table 5, Figure 6 and Figure 7.

Khan et al.^13^ and John et al.^2^ measurements confirmed that calculating the S_cp_ based on the corrections using the OAR(r) 40 × 40 cm^2^ field, leads to errors in the output factors greater than 5% when *r* > 10 cm. In this research, the errors were less, especially at the energy 18 MV, even though the distance *r* was up to 15 cm. Possibly, this is because in the previous studies a homogeneous field in all directions was considered (i.e., no difference between left and right, or up and down field halves), and OAR(r) values were derived from the cross‐beam or diagonal profile only, regardless of the direction of the EFC.

Das et al. ^36^ who studied the applicability of the GESF concept for rectangular fields ranging from 0.5 × 0.5 cm^2^ to 5 × 5 cm^2^, exhibited that differences in field output factors exist between linacs from two different vendors, because of the different design of the collimators. Such differences were observed in our study when comparing S_cp_ values between two Varian Linacs, especially for 3 × 3 cm^2^ fields (see Figures 4 and 5). However, in the Das et al.^36^ study, no asymmetric fields were included.

In Figure 6c there is a jump in D_f_% for the 5 × 5 cm^2^ field size in the case of SOF (y_1_ = −5 cm) for the 6 MV beams only, which was not observed in Figures 6a,b,d. Since this jump was observed in both Linacs, it may be assumed that is attributed to the design of the FF at the measurement point.

Jackson et al.^23^ confirmed that the differences in MU between manual and TPS calculations for breast radiotherapy increase when these calculations require OAR(r) corrections, and compared 6 MV and Cobalt beams (the OAR correction is neglected for Co‐60) under similar conditions while neglecting influencing factors such as tissue heterogeneities in both calculations. Also, computerized investigations proved that these differences are small at the IC and large away from it under the same conditions.^3^, ^24^, ^37^ In addition, Ian et al.^25^, ^26^ proved, after correcting the missing tissue in breast, that these differences increase with the decreasing energy of the photon beam.

Part of the remaining unresolved difference in MUs can be explained by the fact that the equivalent field in the TPS is defined as the symmetric field in which the PDD of its central axis has the same properties as the PDD in the effective axis of the asymmetric field, and this also applies to the S_cp_. This means that the effect of the OAR(r) is included in the TPS calculations, whereas in manual calculations the OAR(r) effect is only corrected for PDD, TPR or TMR and is not taken into account for correcting the output factor or total S_cp_ which are computed directly from the GESF.

In breast radiotherapy with a single‐isocenter technique, after correction of the missing tissue, the GESF for the half‐ or quarter‐ fields does not exceed 10 × 10 cm^2^ and also (*r*) does not exceed 7 cm in most clinical cases,^25^, ^26^, ^38^ so the difference is within 1% (Table 5). This also applies to cases of the brain and head & neck radiotherapy. With respect to craniospinal radiation, the width of the lower spine half‐field is proportional to cover the vertebral bodies only, and therefore *r* and GESF are within the acceptable limits. For more complex clinical cases when radiotherapy is without half‐beam blocking where the EFC can be 10 cm away from the IC and the GESF to the supraclavicular field (off‐isocenter field) is up to 10 × 10 cm^2^,^9^ the agreement is within 2% (Table 5).

In this paper, we have calculated the differences in total S_cp_ between different ASF_s_ and their GESF_s_, and the results can be used for manual MU check calculations of three‐dimensional conformal radiotherapy (3D‐CRT) plans to improve their accuracy. Based on these findings, it is preferable in treatment plans to have the EFC as close as possible to IC, to keep the difference D_f_% as low as possible, especially for the breast radiotherapy when chest wall is longer than 20 cm (single‐isocenter technique without the use of half beam blocks),^9^ as in the case previously shown in Figure 1c.

## 5 CONCLUSION

The results of this study suggest that in cases where the IC is partially or completely blocked, the S_cp_(EFC) is dependent on the distance of the measuring point (i.e., the EFC) from the IC but independent of the method of jaw setting (half‐, quarter‐ and off‐isocenter fields). For all measurements, the S_cp_(EFC) was larger than S_cp_(IC). This increase was up to 2% and 4% for energies 23/18 and 6 MV respectively, but generally it was within 2% for most clinical cases. In this study we have provided detailed information about S_cp_(EFC) measurements and calculations which are tabulated in Tables 1, 2, 3, 4 and can be utilized as correction factors to improve the accuracy of quick, manual verifications of MU in the 3D‐CRT treatment plans, which is still used in some developing countries.

## AUTHOR CONTRIBUTIONS

Mohammad Samir Hmodi, Majeda Nahili and Ousamah Anjak conceived this project. Mohammad Samir Hmodi and Ali Hasan designed the experiments and performed the measurements. Mohammad Samir Hmodi and Karlos Shamout wrote the draft manuscript. Majeda Nahili, Ioannis A Tsalafoutas and Mohammad Hmodi analyzed the data, interpreted the results, and revised the manuscript. All authors have approved the manuscript's final version.

## CONFLICT OF INTEREST STATEMENT

The authors have no conflict of interest to declare.
